# Using novel Canadian resources to improve medication reconciliation at discharge: study protocol for a randomized controlled trial

**DOI:** 10.1186/1745-6215-13-150

**Published:** 2012-08-27

**Authors:** Robyn Tamblyn, Allen R Huang, Ari N Meguerditchian, Nancy E Winslade, Christian Rochefort, Alan Forster, Tewodros Eguale, David Buckeridge, André Jacques, Kiyuri Naicker, Kristen E Reidel

**Affiliations:** 1 Department of Epidemiology, Biostatistics and Occupational Health, McGill University, 1020 Pine Avenue West, Montreal, QC, H3A 1A2, Canada; 2 Clinical and Health Informatics Research Group, McGill University, 1140 Pine Avenue West, Montreal, QC, H3A 1A3, Canada; 3 Department of Medicine, Royal Victoria Hospital, McGill University, 687 Pine Avenue West, Room A3.09, Montreal, QC, H3A 1A1, Canada; 4 Ottawa Hospital Research Institute, 725 Parkdale Avenue, Ottawa, ON, K1Y 4E9, Canada; 5 Collège des Médecins du Québec, 2170 Réné-Lévesque Boulevard West, Montreal, QC, H3H 2T8, Canada

**Keywords:** Adverse events, Medication reconciliation, Transitions in care

## Abstract

**Background:**

Adverse drug events are responsible for up to 7% of all admissions to acute care hospitals. At least 58% of these are preventable, resulting from incomplete drug information, prescribing or dispensing errors, and overuse or underuse of medications. Effective implementation of medication reconciliation is considered essential to reduce preventable adverse drug events occurring at transitions between community and hospital care. An electronically enabled discharge reconciliation process represents an innovative approach to this problem.

**Methods/Design:**

Participants will be recruited in Quebec and are eligible for inclusion if they are using prescription medication at admission, covered by the Quebec drug insurance plan, admitted from the community, 18 years or older, admitted to a general or intensive care medical or surgical unit, and discharged alive. A sample size of 3,714 will be required to detect a 5% reduction in adverse drug events. The intervention will comprise electronic retrieval of the community drug list, combined with an electronic discharge reconciliation module and an electronic discharge communication module. The primary outcomes will be adverse drug events occurring 30 days post-discharge, identified by a combination of patient self-report and chart abstraction. All emergency room visits and hospital readmission during this period will be measured as secondary outcomes. A cluster randomization approach will be used to allocate 16 medical and 10 surgical units to electronic discharge reconciliation and communication versus usual care. An intention-to-treat approach will be used to analyse data. Logistic regression will be undertaken within a generalized estimating equation framework to account for clustering within units.

**Discussion:**

The goal of this prospective trial is to determine if electronically enabled discharge reconciliation will reduce the risk of adverse drug events, emergency room visits and readmissions 30 days post-discharge compared with usual care. We expect that this intervention will improve adherence to medication reconciliation at discharge, the accuracy of the community-based drug history and effective communication of hospital-based treatment changes to community care providers. The results may support policy-directed investments in computerizing and training of hospital staff, generate key requirements for future hospital accreditation standards, and highlight functional requirements for software vendors.

**Trial registration:**

NCT01179867

## Background

Up to 7% of admissions to acute care hospitals are related to adverse drug events (ADEs) [1]. ADEs are the sixth leading cause of death [2] at a cost over $5.6 million (USD) per hospital per year [3]. An estimated 19% to 23% of inpatients will have an adverse event within 30 days of hospital discharge [4,5], 14.3% will be readmitted [6], and 70% of these events will be related to prescription medication [4,5]. Fortunately, at least 58% of these ADEs are preventable, resulting from incomplete drug information, prescribing or dispensing errors, and overuse or underuse of medications [7,8]. Reconciliation of changes to medications that occur during hospitalization with community-based prescriptions is believed to be important to reduce the risk of preventable ADEs during transitions in care. Indeed, one recent study suggests that inadvertent discrepancies in community and hospital medications may increase the risk of adverse events [9]. Hospitals in Canada and the United States now require implementation of medication reconciliation for accreditation [10-13]. Discharge reconciliation has been given the highest priority because it is expected to reduce the risk of adverse events caused by failures to reconcile the community drug regimen with changes made in drugs and doses during the hospital stay. It will also communicate information about medication changes to the responsible community-based pharmacists and physicians at discharge. Despite its importance, there are considerable challenges to widespread implementation [14-31].

### Challenge 1. Obtaining an accurate community-based medication list

Difficulty in obtaining accurate information about the community-based drug list is one of the greatest challenges in medication reconciliation [32]. In a recent survey of hospital staff, respondents estimated that 87% of admitted patients did not know which medications they took, 80% of the time medication information was not available from alternate sources such as relatives or community-based care providers, and in 63% of admissions, hospital staff were unable to access community-based records [32]. As a result, 46% to 67% of unintended discrepancies in medication reconciliation are omitted medications, that is medications that were taken in the community but were neither prescribed at admission nor reconciled at discharge [16,28,29,33-36]. The most commonly omitted medications are cardiovascular drugs, pain medications, anti-infectious medications, and central nervous system medications such as antidepressants and sleeping pills [37]. Overall, 23% to 37% of unintended discrepancies between community and hospital medication are considered clinically significant, meaning that there is substantial potential to cause harm [16,28,29,33-36].

An increasing number of hospitals are employing pharmacists in the emergency department and inpatient units to obtain a complete history of community-based medications [10-12,16-18,20,38-44]. Pharmacists have been shown to be more effective than nurses or medical staff in obtaining an accurate medication history, reducing errors from 323 to 86 per 1,000 prescription orders, compared with nurse-taken histories where errors were reduced to only 157 per 1,000 [37]. The superiority of pharmacists in medication history-taking may be related to two aspects of care. First, pharmacists spend an average of 12.9 minutes per patient to take a community-based medication history, two to three times longer than medical or nursing staff [45]. Second, pharmacists dispense medication and in general are much more knowledgeable about medication characteristics. This expertise may be particularly useful when patients are attempting to recall their medication, as most patients remember their medications by the colour, shape and general purpose of the pill [27,46]. Pharmacists may be more likely to identify these medications than medical and nursing staff, who know the name but not usually the colour and shape of the pill. Indeed, a recent pilot study performed in a US Veterans Affairs hospital found that the integration of pill image files with medication lists was a useful approach to verify current use with patients [27].

Although pharmacist deployment in clinical care areas is considered a cost-effective investment in preventing medication errors [47], pharmacists are conventionally not available on weekends, evenings and nights, nor are community-based pharmacies or office-based practices usually open to transmit information about community-based medications by fax or telephone. As such, recent research has shown that unintended errors in reconciling community and admission medications at discharge are more likely to occur on night-time admission, particularly for elderly patients and those using more than four medications [26].

New initiatives have been undertaken to use electronic medical records to access information about the community drug profile [29-31,48]. Brigham and Women’s Hospital in Boston has shown that retrieval from electronic medical records can identify 65% of current medications [30]. The major limitations of using medication lists in electronic medical records is that many of the listed medications (up to 70%) are no longer being used by the patient as medication lists become out-of-date, and 15.5% of current medications are not listed in the electronic medical record [48]. In contrast, almost all pharmacies have been computerized so that they can manage the online adjudication processes of public and private drug insurance programs [49]. Prior research has shown that records of dispensed prescriptions can be used to accurately measure medication adherence [50-52]. A recent study from the Netherlands also suggests that community pharmacy records can identify up to 97.6% of community-based medications accurately [29]. Although it represents a promising approach, the utility of community-based pharmacy records for medication reconciliation at hospital discharge has not been formally assessed.

### Challenge 2. Ensuring medication reconciliation is conducted for all patients at risk

In compliance with accreditation standards, most hospitals have instituted a paper-based medication reconciliation process. However, adherence is poor, with medication reconciliation generally conducted in less than 20% of patients at risk [10-12,16-18,20,38-44]. This low rate of utilization persists even when staff workload is reduced by an electronic ‘copy and paste’ process that eliminates the need to first document the community-based medication list and then re-transcribe the list for the hospital medication order [26,53]. One of the main barriers is the time and resources required for data collection (community drug list determination), particularly in emergency departments (ED), where most patients are admitted. For a typical ED with 50,000 visits per year, it is estimated that an additional 2,900 hours of nursing time and 8,750 hours of pharmacist time would be required (an added cost of $349,500 at $30/hour) to complete the admission medication reconciliation for the 35% of patient visits where it is required [54]. Moreover, 20% of patients die or are discharged before complete information can be obtained about the community drug list [54].

Overcoming inefficiencies in obtaining the community drug list appears to be essential to improve adherence. For example, when Brigham and Women’s Hospital established a prototype medication reconciliation module that integrated data from the ambulatory electronic medical record and discharge medication orders, they improved adherence to 68.7%, as the majority of physicians could reduce the time to complete the process by 10 minutes. Even higher rates of adherence - from 20% to 90% at admission and 95% at discharge - were achieved at Bellevue Hospital in New York, when admission and discharge orders were blocked until the medication reconciliation module was completed [53]. However, this option is only possible in hospitals that have successfully implemented computerized prescriber order entry, which represents less than 20% of hospitals in the United States and even fewer in Canada [55-57].

### Challenge 3. Communicating drug or dose changes at discharge to community-based prescribing physicians and dispensing pharmacists

A substantial proportion of ADEs occur in hospitalized patients shortly after discharge [4,5]. It is estimated that 72% of medication reconciliation errors at discharge are due to an incomplete preadmission community drug list, while 26% are due to failures in reconciling the medication history or changes made during the hospital stay with discharge orders [36]. During hospitalization, 31% of patients will have changes made in the dose and frequency of medication, 9% will have a medication added or substituted and 4.1% to 8% will have a medication stopped [36,58]. At the present time, there is no timely and effective mechanism of communicating these changes in medication to the community-based prescribing physician(s) and dispensing pharmacist(s). Most patients fill their discharge medication prescription within the first few days after hospital discharge [59], long before the discharge summary that summarizes the reasons for hospitalization and changes in medical management has been dictated or transmitted. Indeed, in the majority of admissions, the community-based care team does not receive critical information on the patient’s health status and modified treatment plan post-discharge [60]. As a result, the patient’s community-based pharmacist needs to determine whether remaining refills on community-based drugs are to be added to the discharge prescription or stopped; and whether the dose prescribed on a discharge medication is to be added or replace the existing preadmission medication dose. As the community-based profile is typically incomplete, these issues are usually not addressed in the discharge prescription. To add to the challenges of discharge reconciliation, 70% of elderly patients who use many medications are under the care of a number of prescribing physicians and over 40% of patients will use more than one dispensing pharmacy [61,62]. For all of these reasons, it is not surprising that 17% to 21% of patients will experience ADEs post-discharge, and that the majority of discrepancies in community and hospital medication reconciliation are related to therapeutic duplication (more than one drug from the same class), dose errors, and omitted medication [7,36,63,64].

In summary, effective implementation of medication reconciliation is essential to reduce preventable ADEs occurring at the transitions between community and hospital care. More efficient methods of obtaining the community drug list, an automated order entry process that facilitates re-ordering of hospital- and community-based medications at discharge, and more efficient means of transmitting discontinuation and change orders to community-based pharmacists and physicians are needed.

### Study objective

To determine if an electronically enabled discharge reconciliation intervention that includes electronic retrieval of community drug lists from community pharmacy records; reconciliation of community and hospital drugs at discharge; and communication of treatment changes to the community-based prescribing physicians and pharmacists will reduce the risk of ADEs, ED visits and readmissions in the 30 days post-discharge compared with usual care.

### Pilot study results

To determine if the electronic retrieval of the community drug list would add value to the usual care process, we conducted a pilot study at the McGill University Health Centre. We used an integrated drug management system (MOXXI) developed previously by our research group to provide online access to the Quebec government prescription database, which includes all medications prescribed by community pharmacies [57,62,65-69]. The MOXXI system provides near real-time information (within 24 hours) on dispensed prescriptions from the 1,800 community pharmacies in Quebec, through a secure virtual private network. This network is linked to the prescription claims system of the government insurer (RAMQ). In this pilot study, we assessed whether the community drug profile was able to identify missing medication at admission; the perceived value of electronic retrieval for the treatment team; and the number of community providers who would be affected by a discharge reconciliation and communication intervention. In 91 consecutive patients admitted in 2008 , we showed that electronically retrieved community pharmacy records identified, on average, three additional drugs per patient. For 21% of patients, five or more drugs were identified. Over 90% of physicians and nurses who accessed real-time community pharmacy records believed this information improved the quality and continuity of care. Overall, 72.7% were confident in their ability to use a computer to gain access (even though 29% had limited or no prior computer experience). Access to the community drug profile reduced medication history-taking by 2.5 minutes per patient. Moreover, the challenges for staff in accessing treatment information for traditional medication reconciliation were substantial: 31% of patients had more than one dispensing pharmacy, most had multiple prescribing physicians, and 14.3% had more than eight (Table 1).

**Table 1:** The number of prescribing physicians and dispensing pharmacies for 91 consecutive patients admitted to the McGill University Health Centre (April-May, 2008)

**Number of prescribing physicians**	**N (%)**	**Number of dispensing pharmacies**	**N (%)**
One	6 (6.6%)	One	60 (69.0%)
Two to four	34 (37.4%)	Two	18 (20.7%)
Five to eight	38 (41.8%)	Three or more	9 (10.4%)
Nine or more	13 (14.3%)		

## Methods/Design

### Trial design

**Figure 1:**
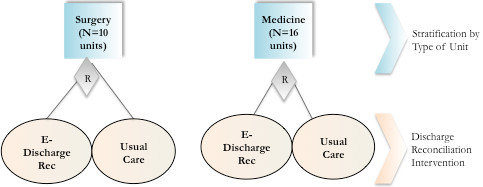
Stratified random cluster design of electronically enabled discharge reconciliation versus usual care.

A randomized cluster design will be used to determine if electronically enabled discharge reconciliation reduces adverse events post-discharge. The study will be conducted at the McGill University Health Centre, a network of teaching hospitals that serves a population of 1.3 million. The study population will be patients admitted to medical and surgical units. Patients will be stratified by type of unit, and a cluster randomization approach will be used to allocate the 16 medical and 10 surgical units to discharge reconciliation and communication versus usual care (Figure 1). A cluster randomization approach is required to avoid contamination between the two interventions (usual care versus discharge reconciliation and communication). This is because all medical staff clustered within units will be trained and provided with onsite support to successfully use computerized order entry for community and inpatient drug reconciliation. It will not be possible to randomize patients within a unit without risk of contamination.

### Participants

Patients will be eligible for inclusion if they are using prescription medication at admission, covered by the Quebec drug insurance plan (50% of the population and 100% of persons 65 years of age and older), admitted from the community, 18 years or older at admission, admitted to a general medical or surgical unit or intensive care, and discharged alive. Patients who are cognitively impaired or otherwise unable to provide consent will be included as we have shown that this subpopulation of patients may be at greatest risk of adverse events because of communication problems [70]. Tri-council ethics guidelines deem that this subpopulation should not be excluded from interventions that potentially provide direct benefit to the participant due to the inability to provide informed consent (Article 4.5) [71]. For these patients, the family or medical director of the admitting unit will authorize study participation. Patients who may not be able to consent at the time of admission because they are cognitively impaired or unable to communicate will be followed up by a researcher to determine if the patient is able to provide an informed consent. At that time, patients who are able to provide informed consent and choose not to will be excluded from the study.

Demographic, clinical and health care service use characteristics of the study population will be retrieved from the admission note. Data from provincial administrative databases in the year before admission and the two months after discharge will be used to characterize the study population, assess readmission, evaluate the integrity of randomization, and assess potential biases related to attrition [72,73].

### Intervention

#### Usual care

The community drug list is generally documented at the time of admission. For patients admitted through the ED, the triage nurse and ED pharmacist (weekdays only) are responsible for documenting the community medication list in the chart. This may be reviewed and updated by the admitting physician, resident and nurse. When the patient is admitted directly to the unit, the admitting nurse and staff physician or resident are responsible for documenting the medication history. In addition, there are 14 full-time equivalent pharmacists available on weekdays to provide inpatient clinical pharmacy service support for the medical and surgical units, including intensive care. Unit-based pharmacists may provide assistance in obtaining the community drug history, particularly for more complex medication regimens.

At discharge, the attending physician or resident uses the list of current hospital medication, with or without the community drug list (if available), to prescribe the discharge medication. Similar to other hospitals, the McGill University Health Centre has implemented an electronic health record (OACIS) that integrates all relevant clinical information from the hospital pharmacy, laboratory, diagnostic imaging and consultation reports to be viewed by the treatment team. Active hospital medications can be viewed by accessing the patient’s electronic OACIS record, the medication administration chart or nurse’s kardex. The patient is provided with a written discharge prescription to fill at their community pharmacy, and may or may not receive verbal or written instructions about new medications or community medications. If the community pharmacist has questions about whether they should continue pre-existing medications that are not included in the discharge medication, they ask the patient, and may call the physician or discharging unit of the hospital.

#### Electronically enabled discharge reconciliation and communication

**Figure 2:**
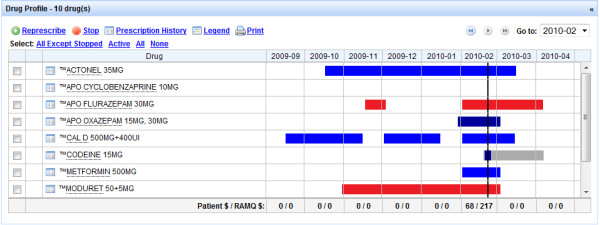
MOXXI drug profile: medication active today (vertical line) and medication history (six months).

**Figure 3:**
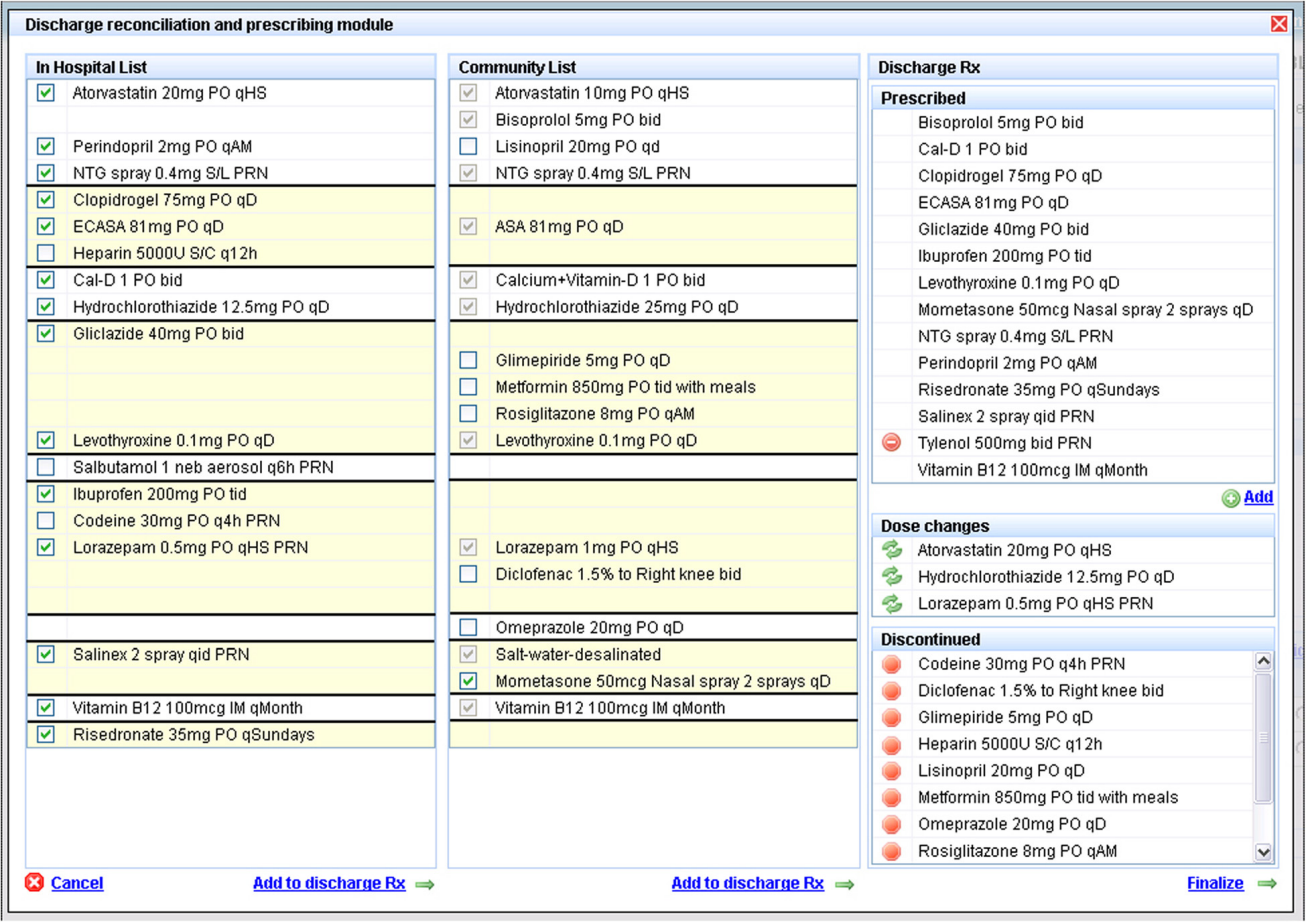
Discharge reconciliation and prescription module.

**Figure 4:**
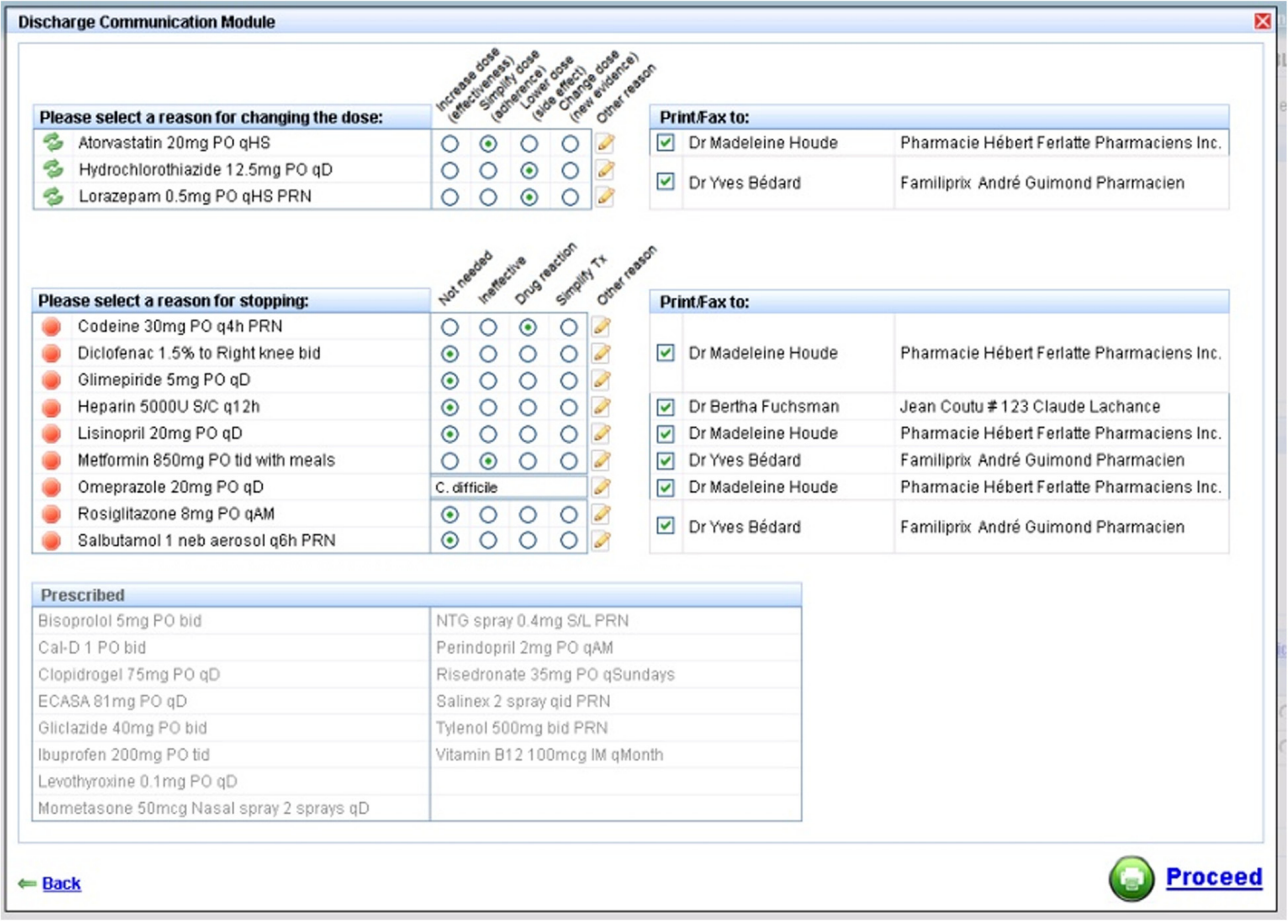
Discharge communication module.

The experimental intervention has three components. First, at admission, the community drug list will be electronically retrieved from the RAMQ using the MOXXI real-time interface, and transferred to the hospital pharmacy system (Figure 2). In a prior validation study, we have shown that RAMQ prescription claims achieve an accuracy of 100% for the drug dispensed, and 98.5% for the date of dispensing [72]. We will include all drugs where the patient has an active supply of medication in the two months prior to admission, as well as provide the treatment team with the option of reviewing all drugs dispensed in the past six months using the MOXXI drug profile. The admitting team and hospital pharmacist will verify the list with the patient, add any other medications including over-the-counter and herbal products, and the resulting list will be used to pre-populate the discharge module (Figure 3). Second, at discharge, the attending physician or resident will write the discharge prescription using the discharge reconciliation module. The discharge reconciliation module will be integrated and directly accessible through the patient’s electronic OACIS record. It will display the current active hospital medications, and the verified community-based drug list, sorted by therapeutic class (for example, antihypertensives, antidepressants) to facilitate reconciliation (Figure 3). The attending physician or resident will ‘click’ on each of the hospital medications that should be included in the discharge prescription, and add any medications from the community drug list that should be continued. All community-based medications that are not included in the discharge prescription will be transferred to the discontinuation section to be verified by the discharging physician. To assist in viewing the results of the reconciliation process, new medication and dose changes will be separated from discontinued medication. Third, the discharge communication module (Figure 4) will facilitate the identification and transfer of information on discontinued and changed medication to the respective dispensing pharmacy or pharmacies and prescribing physician(s) along with the reasons for these changes. The attending physician or resident will document the reason for discontinuing or changing the dose of each medication using a drop-down menu, an approach that has been validated by our group in prior research [65]. A comment field is also available where additional information can be added using free text. The list of prescribed and discontinued medications will be printed, signed by the attending physician or resident, and a copy printed and retained for the chart.

For each discontinued medication or dose change, the dispensing pharmacy and prescribing physician will be identified from the community-based pharmacy claim record. The identity of the prescribing physician and pharmacy must be completed accurately for the pharmacy to receive payment from the RAMQ. Access to this information has been approved for this study by the provincial privacy commission. For each discontinued drug, the name of the patient and drug, the reason for discontinuation, the attending physician, the hospital and the discharge date will be faxed to the respective dispensing pharmacy and prescribing physician.

### Outcome assessment

#### Adverse drug events

An ADE is defined as an injury resulting from medical intervention related to a drug [1,7]. ADEs will be assessed by collecting self-reported patient information 30 days post-discharge; retrieving chart and administrative data on drugs that were started, stopped or continued at discharge as well as acute and chronic health problems; and reviewing and adjudicating the presence of an adverse event and the probability of it being drug-related by a blinded expert panel review of each patient’s chart and post-discharge interview data, using two approaches. The Leape and Bates approach will also be used to assess whether an injury resulting from a drug-related medical intervention occurred, and its severity and preventability [1,7,74]. The Leape and Bates approach will include injuries related to both failing to restart a drug that was held at admission for an elective surgical procedure and injuries caused by unplanned duplications of therapy when changes in treatment occur in hospital without notififying community physicians and pharmacists. The Naranjo criteria [75] will also be used to assess the probability that an event was attributable to a specific drug (that was newly started, changed or continued during hospitalization).

#### Self-report interview

Patient self-report will be used because it is the most sensitive method of ADE detection, identifying an additional 28% of adverse events compared with medical chart review, of which 13% are considered serious [76]. A modification of the Australian two-step adverse reaction and drug event report will be administered by telephone to solicit patient feedback on potential adverse events and their characteristics [77]. In the first step, patients will be asked to report any new health problem or change in their condition since discharge. In the second step, a review of systems is conducted using directed probes for changes in systems-related symptoms or signs that may be drug-related (for example, skin rash, cough). For positive responses, patients will be asked to describe each new problem, and indicate when it started in relation to the initiation, change or termination of drug treatment post-discharge. Most adverse events occur early in the post-discharge period; therefore, we will conduct the follow-up interview in the first 25 to 30 days [7,36]. A trained research assistant will conduct patient follow-up interviews, blinded to intervention status.

#### Chart abstraction

A trained nurse abstractor, blinded to intervention status, will abstract the medical chart data for each patient. Health problems will be coded using the International Classification of Diseases (ICD)-10, medications by generic chemical code using the Anatomic-Therapeutic Classification system, and procedures by the Canadian Classification of Diagnostic, Therapeutic, and Surgical Procedures (CCP). Data will be entered using the MOXXI chart abstraction system as it permits text entries (for example, hydrochlorothiazide 10 mg, rheumatoid arthritis) to be mapped to the respective classification system, and stored by patient identifier in the MOXXI Oracle database. A case summary record that includes the community drug list, hospital drug list, discharge abstract, discharge medications and patient self-report will be prepared for assessment by the expert panel.

#### Expert review and adverse drug events assessment

The chart abstraction case summary and patient self-report interview report will be reviewed independently by three clinicians. Clinicians will be blinded to the patient’s intervention status (usual care versus discharge reconciliation).

First, each clinician will use the Leape-Bates classification system [1,7] to assess whether an ADE was present (yes, no), and its severity into one of four mutually exclusive categories (fatal, life-threatening, serious, significant). Each clinician will also be asked to judge if an event was preventable, based on currently available means, using a four-category Likert scale (definitely preventable, probably preventable, probably not preventable, definitely not preventable). Each clinician will also be asked to note the most likely contributing causes to the event. Inter-clinician agreement is reported to be excellent for judging occurrence (k = 0.98) and preventability (k = 0.92), and moderate for assessing severity (k = 0.32 to 0.37) [1,7].

Second, each clinician will assess the probability that new symptoms or problems occurring post-discharge are related to any one of the drugs that were started, changed or continued at discharge using the Naranjo criteria. The Naranjo criteria for ADE assessment is the most widely used method of adverse drug reaction causality assessment [78]. The Naranjo instrument uses the presence or absence of 10 criteria to assess whether a drug is the cause of an adverse event (for example, renal failure). The relative importance of each criterion is weighted from −1 to +2. The sum of criteria-specific weights is used to classify the probability that the event was drug-related into one of four categories: definite (score ≥ 9), probable (score 5 to 8), possible (score 1 to 4) or doubtful (score ≤ 0) [75]. Inter-rater agreement in assessing events using the Naranjo criteria is good to excellent (k = 0.69 to 0.86). Gold standard ADEs will be defined as those with a causality assessment score classified as definite or probable.

After independent rating, all patient cases will be reviewed and discussed by the panel of three clinicians to reach a consensus classification for both the Leape-Bates classification and Naranjo. We will test inter-rater agreement using 10 training charts before the start of the study, and each month during follow-up using one standardized chart per month. Agreement in scoring on the training and monthly quality control charts will be assessed by an intra-class correlation and by weighted kappa.

#### Emergency department visit or hospital readmission

As a secondary outcome, we will include all visits to the ED or hospital readmission in the 30 days post-discharge, measured using the RAMQ provincial health care databases. This approach ensures that all ED visits and readmissions are included, not just those occurring at the McGill University Health Centre. This is important because ambulances will transport individuals to the closest, open ED or hospital, which often is not the discharging institution. Almost all hospital-based physicians in Quebec are remunerated on a fee-for-service basis [79], and for each medical service delivered, physicians are required to accurately record the treating establishment, and the location of the service (for example, intensive care unit, ED, day hospital, inpatient unit), because location and type of establishment determine the level of remuneration. For each consenting patient, the RAMQ will retrieve all records of services provided in the month after discharge. Patients will be classified as having an ED visit if they have a record of service with a location of a hospital ED, and a readmission if they have a service delivered from an inpatient general hospital unit. In secondary analysis, we will retrieve all ICD-10 diagnostic codes recorded for ED visits and readmissions to provide descriptive information on potential reasons for the visit or readmission.

### Randomization

The 26 hospital units will be assigned a random number, stratified by type (medicine, surgery), and the default random number generator in Statistical Analysis Software (SAS) will be used to randomly assign units within stratum to electronically enabled discharge reconciliation or usual care. There will be approximately 1,857 patients assigned to medication reconciliation at discharge and 1,857 to usual care.

### Sample size

We based our sample size requirements on the difference in the rate of ADEs, as this binary outcome with correlated patient observations required the maximum sample size. For this outcome, we bracketed expected rates of ADEs in the control group based on recent Canadian and US studies [4,5,7] to be between 10% and 19%. We specified an absolute reduction of 5% as the smallest clinically relevant difference that would be worthwhile to detect. A difference of this magnitude would conservatively result in 96,000 fewer ADEs in Canada annually, at an estimated annual cost-saving of $240 million [3]. Specifying an acceptable Type 1 error of 5%, and Type 2 error of 20%, the estimated sample size for the expected range in possible cluster correlations (r = 0.02 to 0.05) varies from 2,852 to 4,423 patient admissions, assuming a baseline rate in the control group of 15% (midway between 10% and 19%). Based on an analysis of readmission rates in 2008, a cluster correlation no greater than 0.03 is expected, which would mean a sample size of 3,376 is required to detect a 5% reduction in ADEs. Based on our prior work, we estimate that 10% of patients will not be reached to complete the post-discharge follow-up interview [4,5]; therefore, we estimate that we will need to recruit 3,714 patients to achieve our sample size requirements. Overall, in 2008, there were 17,480 admissions to the McGill University Health Centre, of which 12,236 were admitted to eligible medical and surgical units. Based on our pilot study, approximately 42% (n = 5,139) of patients will be eligible for inclusion (that is, have public drug insurance, ≥ 18 years old, first admission in study period, alive at discharge), and of these 3% will refuse to participate, 45% will not be asked for consent because the staff are too busy, and 52% will consent to participate. By providing staff with support to obtain consent from patients, we expect that we can increase the proportion of eligible patients participating to 60%, or 257 per month. Assuming 10 months per year of active recruitment (to account for summer and winter holidays), we estimate that it will take 15 months to recruit all patients. With an average length of stay of 8.9 days, we should successfully complete enrolment and follow-up in 18 to 20 months.

### Data management and analysis

Four sources of data will be assembled and linked to address the study objective: abstracted medical chart documentation of patient demographics, admission and discharge dates, the community drug list, discharge prescription, and admitting and discharge diagnoses; adverse events information and assessment post-discharge; the RAMQ medical services and prescription claims data; and co-intervention data collected by the study coordinator. All data will be managed in an Oracle database, and files for individual patients will be linkable through a study identification number and Quebec medicare number, with nominal information retained in a separate encrypted file. The integrity of randomization will be assessed by characterizing the age, sex, baseline number of visits, hospitalizations, medications, and comorbidity of patients using the Charlson comorbidity index, a weighted index of conditions that increase the risk of mortality [80]. The CONSORT guidelines will be followed to document the eligibility and follow-up of patients and inpatient units (clusters) in the trial [81]. Provincial health administrative data will be used to characterize bias related to patients lost to follow-up after discharge for whom ADEs cannot be measured, based on the secondary outcome (readmission or ED visits).

An intention-to-treat approach will be used to analyse study results. To determine whether discharge reconciliation reduces the risk of ADEs post-discharge, we will use logistic regression within a generalized estimating equation framework to account for clustering of patients within unit [82]. The presence of an ADE (either identified as present based on the Leape-Bates classification or Naranjo criteria) will be the outcome variable, the hospital unit will be the defined clustering factor, and an exchangeable correlation matrix will be used to account for clustering of patients within unit. Discharge reconciliation will be fit as a dummy variable, using usual care as the reference group. We will use the same approach to assess the secondary outcome, ED visits or hospital readmission. For both the primary and secondary outcome, we will assess whether adjustment for co-interventions and baseline differences between patients in the usual care and intervention arm confound the effect of the intervention. In a secondary analysis, we will assess whether the effect of the intervention is modified by hospital unit type (medicine versus surgery) or patient characteristics that are associated with a higher risk of adverse events (age, number of medications at discharge) by including respective interaction terms in the logistic model and testing their significance using the Wald chi-square statistic.

### Bias and blinding

Our main challenge in bias control is the inability to blind staff to treatment assignment, and potential co-intervention. To control bias, the research assistant, expert panel and analyst assessing the outcome of treatment will be blinded to unit and treatment allocation status. To assess bias related to possible co-interventions, the project coordinator will conduct a monthly review with the unit directors and hospital pharmacy to assess co-interventions (new initiatives that may modify the study outcomes), and we will use sensitivity analysis to assess the potential impact on the study outcomes. As the involvement of the hospital pharmacist in the patient’s care likely reduces the risk of adverse events (for example, through medication review), we will retrieve this information from the chart and assess whether hospital pharmacist intervention (yes versus no) is a confounder by including this information as a patient-level variable in the analysis. In addition, as cluster randomization may not produce patients groups who have an equivalent risk of adverse events post-discharge (that is, because randomization is by unit rather than by patient), we will assess whether patients admitted to the usual care versus electronically enabled discharge units had a similar rate of hospitalizations, and ED visits in the 12 months prior to admission using RAMQ medical service data retrieved for each patient. In addition, we will include prior ED or hospitalization history in the analysis to determine if it confounds the estimated effect of the intervention.

### Ethical considerations

This study received full board review from the McGill University Health Centre Research Ethics Board and was found ethically acceptable for conduct on March 9 2011.

To monitor any unintended adverse effects arising from the study intervention(s), we will establish an independent data monitoring board, chaired by Dr David Bates (Harvard University), and including Dr Gordon Schiff (Harvard University) and Dr Anne Holbrook (McMaster University), scientists who provide pre-eminent expertise in ADEs and computerization of health care. The data monitoring board will monitor quarterly statistics on hospital readmission and ED visit rates to assess unintended effects, blinded to study group. The study team will be responsible for providing any additional statistical information that is deemed necessary by the data monitoring board to ensure patient safety.

## Discussion

A major challenge in this study will be to ensure that attending physicians or residents have sufficient training, motivation and support to use the medication reconciliation module for the discharge prescription. The four conditions needed for successful adoption will be incorporated into the implementation of discharge reconciliation [83]. First, we will train local leadership within each unit to champion the discharge reconciliation process, comprising the unit service chief, head nurse and liaison pharmacist. Second, we will pre-test and calibrate the user interface to minimize workflow disruption and maximize efficiency gains. Based on our experience of instituting electronic prescribing in the primary care setting, we expect that we can save three or more minutes per discharge prescription by allowing relevant hospital and community-based prescriptions to be copied to the discharge prescription [68]. Third, we will prepare, with the clinical champions, a blitz launch in each unit that will feature onsite support for completing the discharge prescription in each unit for the first two weeks. We have successfully used nursing and medical students, who are typically adept computer users, to provide real-time coaching and support for using new clinical computer applications. Last, we will monitor adoption by using application audit trails, and use this information to identify and remedy problems that may exist on certain units.

Medication reconciliation at hospital discharge is expected to reduce unintended discrepancies in community- and hospital-based treatment, and minimize preventable ADEs. We expect that the intervention evaluated in this trial may improve adherence to medication reconciliation at discharge, the accuracy of the community drug history, and effective communication of hospital-based treatment changes to community-based care providers. The solutions we will test are available in all Canadian provinces and many other countries, and made accessible to hospital- and community-based care teams. If we find that the intervention reduces ADEs, it will support policy-directed quality investments in computerization and training hospital staff to use pharmacy-based records and a discharge reconciliation module to improve medication reconciliation. It will also generate key requirements for medication reconciliation that can be applied in future hospital accreditation standards, as well as highlight functional requirements for software vendors.

### Trial status

Anticipated start date: September 2012.

## Competing interests

The authors declare that they have no competing interests.

## Authors’ contributions

RT, AH, AF, AM, NW, AJ, CR and DB conceived of the study and participated in its design and oversight. KN and KR participated in coordination and interpretation of results, as well as manuscript writing. All authors read and approved the final version of the manuscript.
